# Zinc deficiency is highly prevalent and spatially dependent over short distances in Ethiopia

**DOI:** 10.1038/s41598-021-85977-x

**Published:** 2021-03-22

**Authors:** Adamu Belay, Dawd Gashu, Edward J. M. Joy, R. Murray Lark, Christopher Chagumaira, Blessings H. Likoswe, Dilnesaw Zerfu, E. Louise Ander, Scott D. Young, Elizabeth H. Bailey, Martin R. Broadley

**Affiliations:** 1 Center for Food Science and Nutrition, Addis Ababa University, P.O.Box 1176, Addis Ababa, Ethiopia; 2 Food Science and Nutrition Research Directorate, Ethiopian Public Health Institute, Gulele Sub City, P.O. Box 1242, Addis Ababa, Ethiopia; 3 Faculty of Epidemiology and Population Health, London School of Hygiene and Tropical Medicine, Keppel Street, London, WC1E 7HT UK; 4 School of Biosciences, University of Nottingham, Sutton Bonington Campus, Loughborough, Leicestershire, LE12 5RD UK; 5 Department of Public Health, School of Public Health and Family Medicine, College of Medicine, University of Malawi, Chichiri, Private Bag 360, Blantyre 3, Malawi; 6 Inorganic Geochemistry, Centre for Environmental Geochemistry, British Geological Survey, Nottingham, NG12 5GG UK

**Keywords:** Biochemistry, Biomarkers

## Abstract

Zinc (Zn) is an essential nutrient for human health. In Ethiopia, a high prevalence of Zn deficiency has been reported. To explore demographic variation and spatial dependencies in the Zn status of the Ethiopian population, we analyzed archived serum samples (n = 3373) from the 2015 Ethiopian National Micronutrient Survey (ENMS), a cross-sectional survey of young children, school-age children, women of reproductive age (WRA) and men conducted in all 9 regions and two city administration of Ethiopia. Serum Zn concentrations, measured using inductively coupled plasma-mass spectrometry (ICPMS), were compared to thresholds based on age, sex, fasting status, and time of blood collection, after adjusting for inflammation status. Median serum Zn concentration of the population was 57.5 μg dL^−1^. Overall, it is estimated that 72% of the population was Zn deficient, with high prevalence in all demographic groups. Spatial statistical analysis showed that there was spatial dependence in Zn status of WRA at distances of up to 45 km. Zinc deficiency is spatially dependent over short distances. Although WRA in most areas are likely to be Zn deficient, prevalence of deficiency varies at regional scale and between rural and urban inhabitants, suggesting there is scope to explore drivers of this variation, prioritize nutritional interventions, and to design more representative surveillance programs.

## Introduction

Zinc (Zn) is essential for normal immune function, and physical growth^1,2^ and the structure and activity of more than 200 enzymes^3^. Micronutrient deficiencies, including Zn, are widespread in sub-Saharan Africa (SSA), where they remain a major public health problem^4^. Zinc deficiency affects 17% of the world’s population with the highest estimate in Africa (24%) and Asia (19%) based on dietary supply estimates^5,6^. Deficiency of Zn compromises the immune system^7^, resulting in increases in morbidity and mortality during early childhood and is associated with an increased risk of impaired growth and cognition^7–10^.

There are few data on population Zn status in many areas of the world, including most of SSA^11^. The prevalence of Zn deficiency at national scales in Africa has been estimated indirectly, based on national/regional food composition and food supply data, revealing that ~ 75% of the population of Eastern Africa and 81% of the Ethiopian population could be at risk of Zn deficiency due to inadequate dietary Zn supply^11^.

A more direct measure of population Zn status is based on blood plasma or serum Zn concentration relative to thresholds specific to age, sex and time of day. Micronutrient surveillance studies collect a range of micronutrient data from a proportion of the population, in order to make estimates of nutritional status across the represented demographic groups, and are widely used by national public health institutions to track changes in population status, including those which may be due to programmatic interventions, such as targeted supplementation or food fortification. In Ethiopia, the estimated pooled prevalence of Zn deficiency among pregnant women, from a systematic review of seven studies, was 59.9% (95%CI: 51.6, 67.7%)^12^ and the estimated pooled prevalence of Zn deficiency among children from six studies in Ethiopia was 38% (95%CI: 22.9, 55.7%)^12^. This high prevalence of Zn deficiency is consistent with reports of low dietary Zn intake. For example, Girmay et al. reported that the mean dietary Zn intake among children 6–35 months (n = 6,752) was 1.74 mg day^−1^ at national level^13^, which is small compared to an Estimated Average Requirement (EAR) of 2.5 mg day^−1^. Furthermore, the phytate contents of Ethiopian diets are high which is likely to inhibit Zn absorption^5^.

Deficiency of micronutrients for different demographic groups have been reported in the most recent Ethiopian National Micronutrient Survey (ENMS), including for Zn^14^, and also in Belay et al. for selenium (Se)^15^. This survey is a cross-sectional study, which represents all 9 regions and two city administration of Ethiopia. The data reported in those studies were not adjusted for inflammation status, yet it is known that the presence of inflammation or infection affects the assessment of Zn status due to a metabolic redistribution of Zn from the plasma to the liver^6,15–17^. Inflammation biomarkers, including C-reactive protein (CRP) and α-1-glycoprotein (AGP), are recommended to consider when estimating Zn deficiency^10^, because they provide a measure of the severity and duration of inflammation, respectively^18^. In the most recent National Micronutrient Survey in Malawi, estimates of the prevalence of Zn deficiency decreased from 62 to 52% when data were adjusted for inflammation status^19^. In a survey of thirteen countries, not including Ethiopia, the estimated prevalence of Zn deficiency decreased by a median of 11 (range: 4–18) percentage points, compared with unadjusted prevalences^20^. In addition to inflammation, the ENMS has not yet explored spatial dependencies, beyond regional or district aggregations, as seen for Se status at distances of up to 200 km for WRA^15^.

The aim of the current study was to explore Zn status among different demographic groups of the Ethiopian population, and to use spatial statistical models to determine spatial dependencies in Zn status for WRA. Serum Zn concentrations were measured in archived samples from the ENMS using ICPMS, which is a more sensitive analytical method than those reported previously. Inflammation markers, measured in the original ENMS programme, were used for adjustment based on established methods^19,21^. Geostatistical methods, used previously for Se^15^ and which include explicit assumptions, were applied to model the spatial variation of serum Zn concentration and predict the unsampled area across the country, using data for WRA, the demographic group with the largest sample size in the ENMS. These results provide novel and comprehensive information on the prevalence and sub-national variation of population Zn status in Ethiopia. The data contribute to our understanding of the public health significance of Zn deficiency, can inform the prioritization of potential interventions to alleviate Zn deficiency.

## Results

### Characteristics of the ENMS study participants

The largest number of participants in the ENMS was drawn from Oromia Region (16.9%) and the smallest number from Addis Ababa (4.8%) Administrative city (Table 1). Among 3,064 observations with complete information, 16.9% were Young Children (YC), 31.4% were School-Aged children (SAC), 13.1% were men and 38.6% were WRA. About a quarter of the participants were from urban areas. Around half of the SAC (n = 429/962) and YC (n = 283/519), respectively, were male. Half of the household heads were literate (Table 1).

**Table 1:** Characteristics of study participants in the Ethiopian National Micronutrient Survey (ENMS), 2015.

Characteristics	N	Percentage
**Regions**		
Addis Ababa	148	4.8
Afar	270	8.8
Amhara	481	15.7
Benishangul-Gumuz	210	6.9
Dire Dawa	160	5.2
Gambela	192	6.3
Harari	168	5.5
Oromia	517	16.9
SNNPR	392	12.8
Somali	193	6.3
Tigray	333	10.8
National	3064	100
**Demographic group**		
Young children	519	16.9
Male	283	
Female	236	
School age children	962	31.4
Male	429	
Female	533	
Men	402	13.1
Women of reproductive age	1181	38.6
**Residence**		
Urban	768	25.1
Rural	2296	74.9
**Household head education**		
Educated	1499	48.9
Illiterate	1565	51.1
**Inflammation stage**		
No inflammation	2123	69.3
Incubation	70	2.3
Early convalescence	262	8.5
Late convalescences	609	19.9

Stages of inflammation were categorized as: no inflammation (CRP ≤ 5 mg L^−1^ and AGP ≤ 1 g L^−1^); incubation (CRP > 5 mg L^−1^ and AGP < 1 g L^−1^); early convalescence (CRP > 5 mg L^−1^ and AGP > 1 g L^−1^); late convalescence (CRP ≤ 5 mg L^−1^ and AGP > 1 g L^−1^) based on Thurnham et al. (2010)^21^.

### Serum Zn concentrations

The estimated population mean serum Zn concentration is 57.7 μg dL^−1^ (median of 57.5 μg dL^−1^) following adjustment for inflammation. Estimated population serum concentrations vary by Region. The Tigray Region had the lowest median serum Zn concentration (55.2 μg dL^−1^) while the highest median serum Zn concentration (68.3 μg dL^−1^) was recorded for participants from Addis Ababa. The disaggregated data of average serum Zn concentration by administrative zones for the larger regions is indicated in Supplementary Table S1. The median serum Zn concentrations varied by demographic groups, with 47.1 μg dL^−1^ observed in YC and 62.6 μg dL^−1^ in men (Table 2). Urban populations had a greater serum Zn concentration than the rural populations. Serum Zn concentration was greater in households whose head was educated.

**Table 2:** Serum Zn concentrations and prevalence of deficiency by study characteristics in the Ethiopian National Micronutrient Survey (ENMS).

Characteristics	Serum Zn (µg dL^−1^)^a^					Prevalence of serum Zn deficiency (%)^b^	
	Unadjusted			Adjusted			
	N	Mean^a^	Median^a^	Mean^a^	Median^a^	Unadjusted^c^	Adjusted^c^
**Regions**							
Addis Ababa	148	67.5	67.9	68.2	68.3	27.2	25.4
Afar	270	59.2	58.6	60.1	59.4	63.2	61.0
Amhara	481	57.6	57.4	58.6	58.1	74.1	71.5
Benishangul-Gumuz	210	57.7	57.6	58.9	58.5	69.9	65.7
Dire Dawa	160	60.9	60.8	61.9	61.7	66.4	64.0
Gambela	192	59.3	58.3	60.4	59.3	64.5	63.2
Harari	168	56.4	55.7	57.3	56.9	71.8	69.7
Oromia	517	54.8	54.5	56.1	55.6	80.0	76.7
SNNPR	392	57.0	57.3	58.4	58.8	73.3	69.8
Somali	193	60.5	59.7	61.8	60.9	66.5	63.8
Tigray	333	54.8	54.0	55.6	55.2	78.5	75.5
National	3,064	56.5	56.2	57.7	57.5	75.1	72.1
**Demographic group**							
Young children	519	47.2	46.1	48.6	47.1	92.1	89.3
School age children	962	56.1	55.9	57.9	57.8	75.8	71.0
Men	402	60.4	61.1	62.0	62.6	73.9	69.5
Women of reproductive age	1,181	59.8	59.6	60.2	60.0	67.3	66.2
**Residence**							
Urban	768	61.8	61.7	62.8	62.7	58.1	55.3
Rural	2,296	55.6	55.4	56.9	56.7	78.0	74.9
**Household head education**							
Educated	1,499	57.6	57.6	58.8	58.7	72.5	69.7
Illiterate	1,565	55.7	55.5	56.8	56.6	77.3	74.1
**Inflammation stage**							
No inflammation	2,123	57.3	57.0	57.9	57.7	72.9	71.4
Incubation	70	56.8	59.4	58.2	60.1	78.2	72.7
Early convalescence	262	53.6	54.8	55.9	56.9	78.2	74.4
Late convalescence	609	55.2	54.3	57.5	56.9	80.7	73.6

^a^The estimation was weighted using the ENMS sampling weight factor.

^b^Zn deficiency was defined based on serum Zn considering age, sex, fasting status and time of blood collection^10^.

^c^BRINDA internal regression correction approach, which accounts for both CRP and AGP, was applied to calculate the prevalence of Zn deficiency (Namaste et al*.* 2017)^22^.

Adjustments for inflammation decreased the estimated overall prevalence of Zn deficiency from 75.1 to 72.1% (Table 2). Those who were affected with inflammation had lower serum Zn status than those who were not affected. The reference values used to adjust for inflammation were > 5 mg L^−1^ for CRP and 1 g L^−1^ for AGP. These values are the cut-off points for determining inflammation^21^ and as such, some observations which are classified as not inflamed were also adjusted for inflammation.

Among the population within this study, 31% had elevated CRP and/or AGP concentrations indicative of inflammation (Table 1). Therefore, exclusion of these groups from the analyses would reduce the sample size and may bias the results, as reported in other studies^7^.

We determined Zn deficiency prevalence across regional and demographic groups before and after correcting serum Zn concentration based on the BRINDA internal regression correction approach^22^. The prevalence of serum Zn deficiency was different across residence categories (55.3% in Urban vs 74.9% in Rural) (Table 2). The highest prevalence of serum Zn deficiency was found in Oromia (76.7%), Tigray (75.5%), Amhara (71.5%) Regions, and the lowest in Addis Ababa (25.4%).

### Correlations among Serum Zn, inflammation biomarker, age and incidence of diarrhea

Serum Zn level was correlated with age of participant (r = 0.24). Serum Zn concentration was negatively correlated with inflammation (AGP) (r =  − 0.14).

### Spatial variation of Zn status

**Figure 1:**
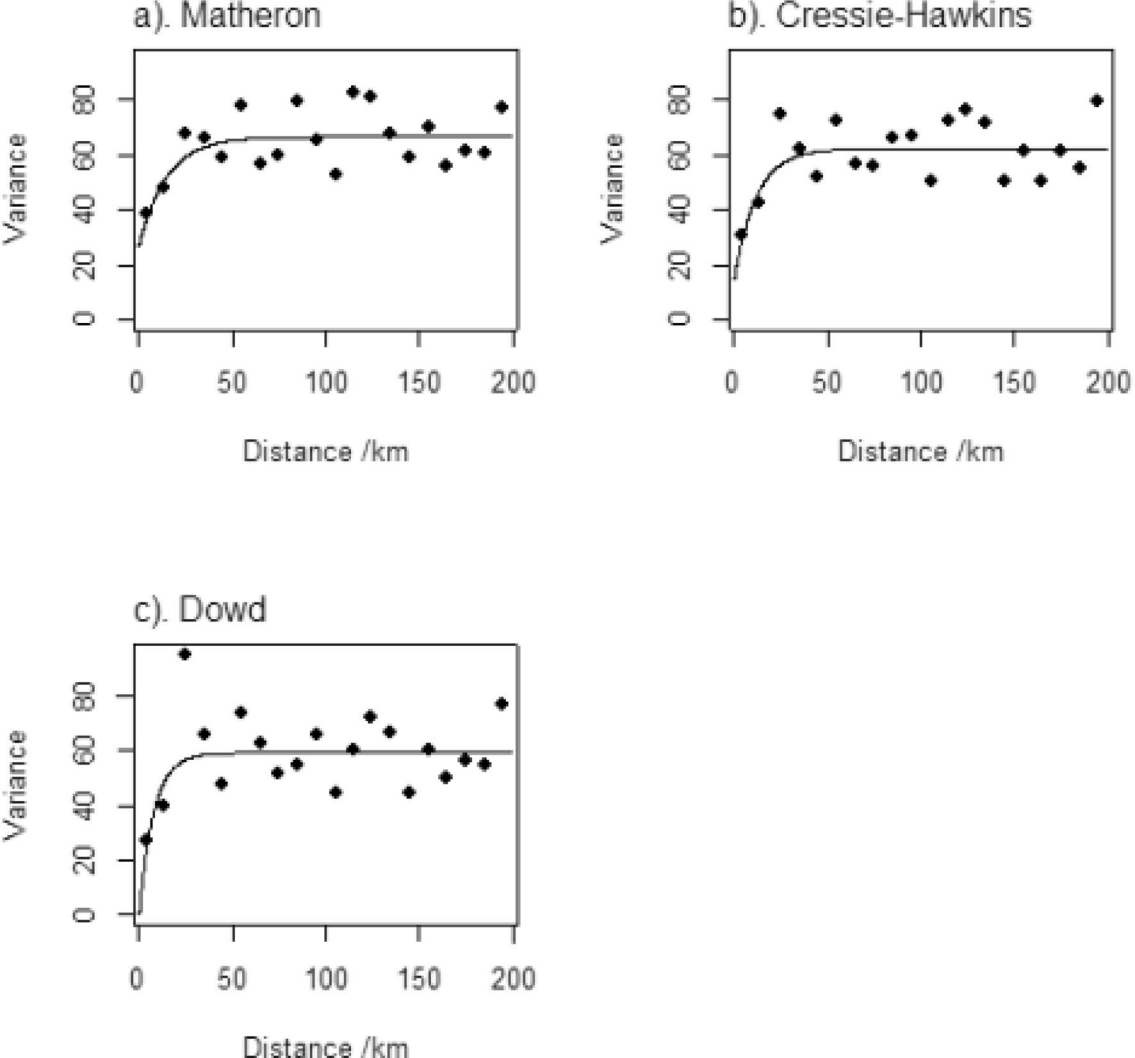
Variograms of WRA serum Zn estimated by different estimators; (**a**) Matheron estimator, (**b**) Cressie–Hawkins estimator and (**c**) Dowd estimators.

The spatial variation of Zn status was explored using the data for WRA, applying methods similar to those described in a previous study on Se^15^. Thus, estimates of the variogram were obtained using alternative approaches, with exponential models fitted by weighted least squares (Fig. 1).

**Figure 2:**
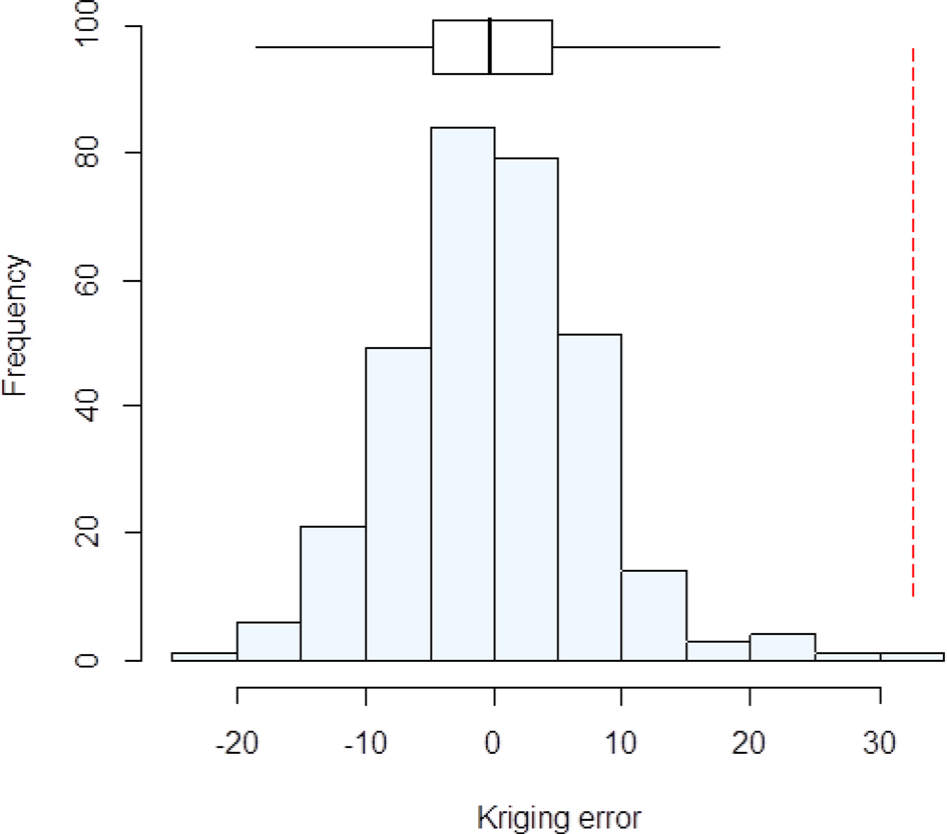
Histogram of the cross-validation errors using the variogram fitted to estimates obtained with the Matheron (1962) estimator^23^. The Box-plot shows the median and quartiles (box limits). The vertical dashed line shows the outer fence values of Tukey (1977)^25^.

The cross-validation errors for the model fitted to the estimates using Matheron (1962)^23^ appear to be normally distributed (Fig. 2), with a median SSPE of 0.414. This model was therefore selected given that the SSPE is close to the expected value (0.455), and it falls within the 95% confidence interval expected value when the model is valid (0.355–0.575). It had a nugget variance component—which describes the variation at finer scales than can be resolved by the sampling scheme, in addition to analytical error of 27.0. The spatially correlated variance was 39.62. The distance parameter, which controls how the spatial autocorrelation diminishes with distance, was 14.79 km.

There is therefore limited spatial dependence in the variation of serum Zn concentration. A large proportion of the overall variance is attributable to the spatially uncorrelated nugget effect, and the correlated variance is spatially correlated up to distances of 45 km, as seen in the variograms^24^, suggesting that Zn nutritional status of WRA in Ethiopia is determined by factors that vary over short distances.

**Figure 3:**
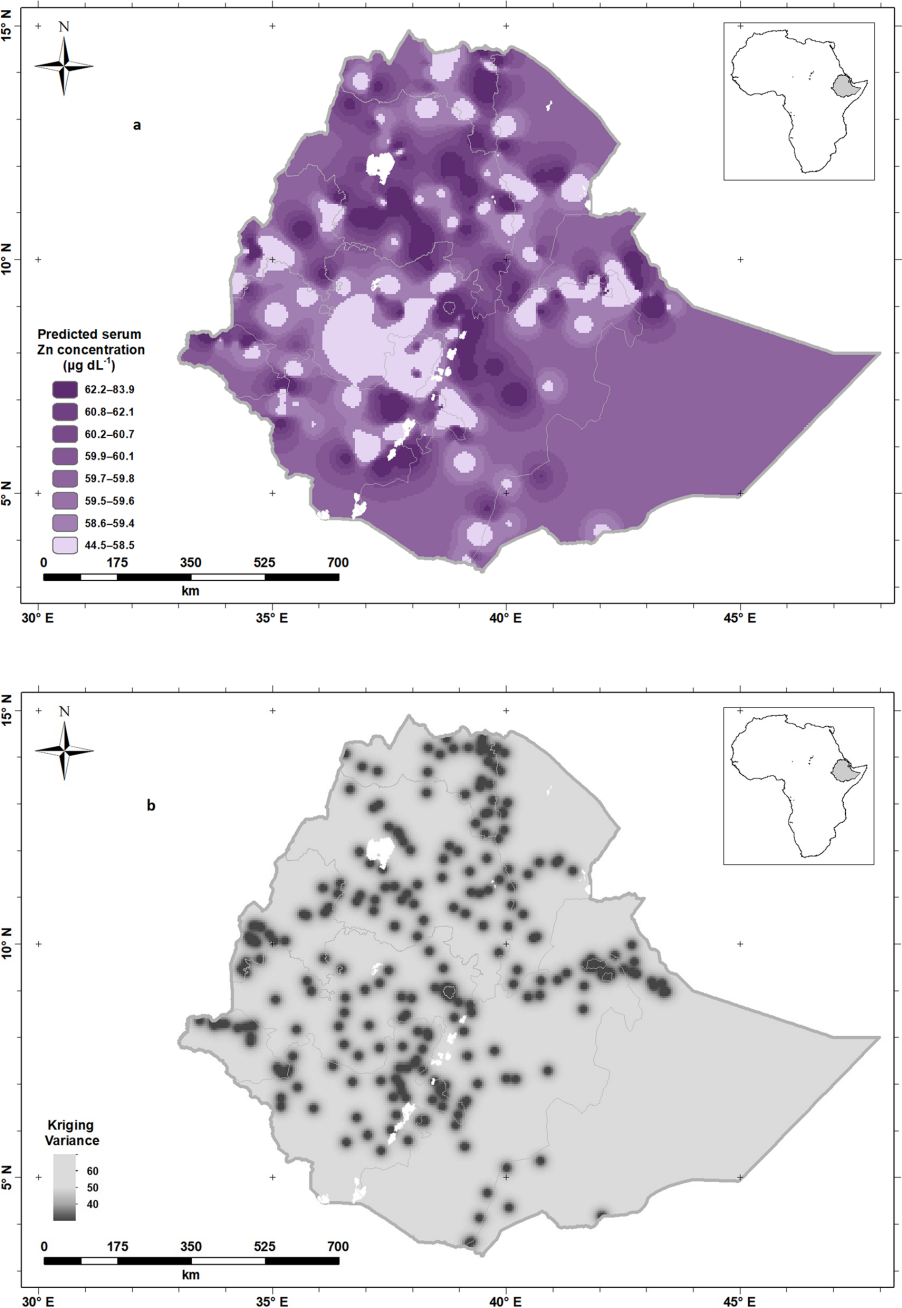
Predicted mean serum Zn concentration (**a**) and Predicted serum Zn variance (**b**), for women of reproductive age in Ethiopia. Created using ArcGIS 10.4.1. ESRI ArcGIS Desktop: Release 10. Environmental Systems Research Institute, Redlands. (2011).

The spatial variation predicted for serum Zn concentration for WRA across Ethiopia obtained by ordinary kriging is shown in Fig. 3a. The larger predicted serum Zn concentrations were found in some areas of the country including Addis Ababa while smaller predicted Zn concentrations were observed in Oromia Region, in line with the observations made on the population-weighted predictions aggregated by Region (Table 2). However, this approach illuminates intra-Region variation which is predicted, based upon the location of the samples. The kriging variances of the predictions of serum Zn concentration for WRA are shown in Fig. 3b; these are the expected squared errors of prediction which are minimized by the kriging prediction based on the variogram model.

As described previously for Se^15^, these interpolation errors are inevitable for all spatial predictions. This is because, given that serum Zn varies spatially, predictions are being made from sparse observations. However, by using the kriging variance (Fig. 3b), we can understand better where spatial predictions are reliable from a particular distribution of observations. It is apparent that there is uncertainty in most parts of the country because of the short distance over which serum Zn is spatially correlated and the sparse distribution of observations^26^. In these conditions, the predicted value tends to the mean of the observations, and the kriging variance is large. Where the kriging variance is large (shaded in light gray colors), further local sampling would be needed to determine Zn deficiency prevalence with greater confidence.

**Figure 4:**
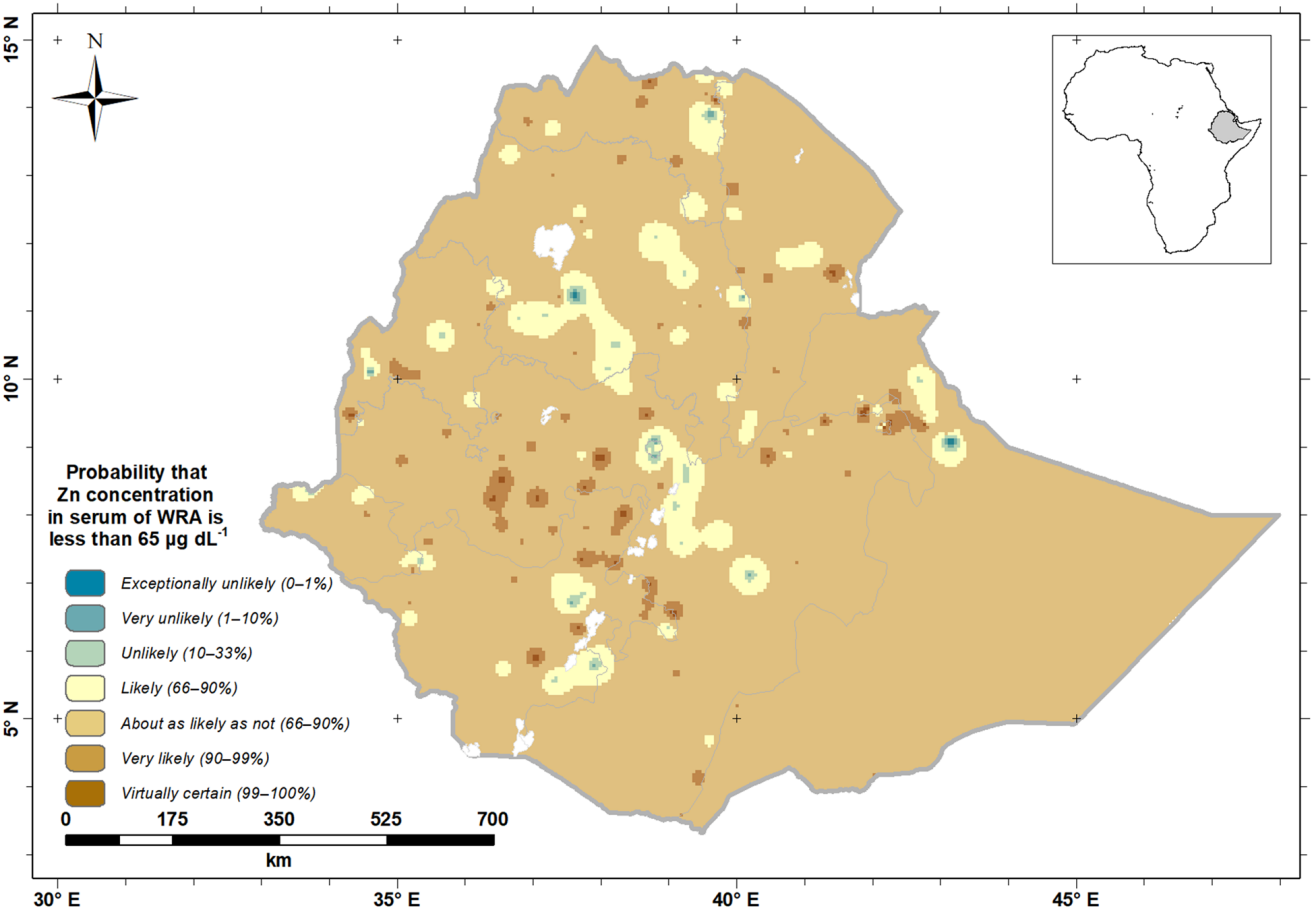
Probability that serum Zn concentration of Ethiopian women of reproductive age (WRA) would fall below a threshold (< 70 μg dL^−1^). Created using ArcGIS 10.4.1. ESRI ArcGIS Desktop: Release 10. Environmental Systems Research Institute, Redlands. (2011).

Figure 4 shows a probability map that the mean serum Zn concentration of WRA is below the cut-off for adequacy (< 70 μg dL^−1^). The map legend includes the “calibrated phases’’ of the Intergovernmental Panel for Climate Change (IPCC)^27^, to improve the communication of uncertainty to all stakeholders^28,29^. This shows that WRA in large parts of Ethiopia are predicted to be ‘likely’ through to ‘virtually certain’ to have deficient serum Zn status (Fig. 4). In central parts of Ethiopia and other small parts of Northern and Eastern Ethiopia, WRA are ‘unlikely’ through to ‘exceptionally unlikely’ to be Zn deficient.

## Discussion

Reanalysis of archived serum samples from the ENMS using ICMPS, with data adjusted for inflammation status, revealed 72.1% of the population of the ENMS were Zn deficient. The median serum Zn concentrations was 57.5 µg dL^−1^, which is low compared to data from other countries^20^, and the lower threshold considered indicative of optimal nutritional status. The highest prevalence of Zn deficiency was in Oromia (76.7%) and Tigray Regions (75.5%); the lowest prevalence was in Addis Ababa (25.4%). Serum Zn concentrations varied between demographic groups with median concentrations greatest among men (62.6 µg dL^−1^) and smallest among YC (47.1 µg dL^−1^). Age was significantly positively associated with serum Zn concentration (r = 0.24) (Table 3). The increase in serum Zn concentration with age is consistent with studies in the USA and France^30,31^.

**Table 3:** Spearman’s rank correlations between serum Zn, inflammation biomarkers, the incidence of diarrhea, and age of all demographic groups.

	Serum Zn (μg dL^−1^)	AGP (g L^−1^)	CRP (mg L^−1^)	Age (years)	Diarrhea
Serum Zn (μg dL^−1^)	1.00				
AGP (g L^−1^)	− 0.14	1.00			
CRP (mg L^−1^)	− 0.05	0.40	1.00		
Age (years)	0.24	− 0.17	0.08	1.00	
Diarrhea	− 0.06	0.07	0.07	− 0.05	1.00

*AGP* α-1-glycoprotein concentration, *CRP* C-reactive protein concentration.

In this study, inflammation was detected among 11% and 28% of participants based on elevated serum CRP or AGP concentrations, respectively (9% of partcipants had elevated concentration of both). Mean serum Zn concentration was highest in participants with no inflammation (57.3 µg dL^−1^) and lowest in participants with inflammation in the early convalescence state (53.6 µg dL^−1^). The overall correlation between inflammation and serum Zn was relatively low (r =  − 0.14 with AGP). The relationship between serum Zn concentration and inflammation biomarkers have not been widely studied^32^. However, it has been noted previously that infection can produce Acute Phase Reactants (APRs) and a decline of serum Zn concentration^17^. In a review of data from micronutrient surveys in thirteen countries, the lowest serum Zn concentrations were found in YC classified as being in the early convalescence state of infection, which is consistent with the finding of our current study^20^.

Along with inflammation, serum Zn concentration is also influenced by time of the day of blood collection in a fasting or nonfasting state. In this study the highest mean (61.7 µg dL^−1^) and median (61.7 µg dL^−1^) concentrations were observed in participants who had blood drawn in the fasting state, followed by samples taken in the morning from those in the non-fasting state. This is consistent with other studies^33^. Therefore, to determine Zn status in populations, inflammation biomarkers and time of the day of blood collection should be taken into consideration to estimate a reliable Zn deficiency prevalence.

The largest prevalence of deficiency was observed in Oromia and Tigray, followed by Amhara Regions. Tessema et al*.* 2018^34^ reported that half of the households in Tigray (50%) and a quarter of households in Amhara (25%) live in areas where soil concentrations of Zn are low, potentially limiting entry of Zn into local diets. In their study, the correlation between serum Zn and soil Zn was significant in Ethiopia, but it was relatively low (r = 0.09), indicating that other determinants affect serum Zn.

The large proportion of the population we have estimated to have inadequate Zn nutritional status is a finding which is consistent with other approaches that have been published. In Ethiopia, a high national prevalence of Zn deficiency has been estimated previously based on Zn supplies in the food system, which reported that 81% of the population were at risk of deficiency due to inadequate dietary Zn supplies, estimated from food balance sheets and regional food composition data^11^. A high prevalence of Zn deficiency (> 20%) has also been reported based on serum Zn status and dietary Zn intake among young children and school-aged children^13,34–36^ and women and pregnant women^12,37,38^. Several factors may contribute to a high prevalence of Zn deficiency in Ethiopia, where the consumption of Zn-rich foods such as meat is low^39^. Food systems are highly localized in Ethiopia, particularly in rural areas, with a large proportion of dietary intakes met through subsistence production or purchases of locally-produced food^40^. Thus, individuals’ Zn status may reflect the soil types and landscapes where they reside.

From the geostatistical analysis, the selected variogram (Fig. 1a, Matheron) and the fitted parameters show that about 60% of the variance is spatially dependent (i.e. the spatially dependent component as a proportion of the sum of the two variance components). However, spatial dependence is limited to about 45 km (i.e. 3 × the distance parameter). That is to say, two observations which are close together (less than 45 km) are more likely to be similar than two observations separated by a greater distance. The correlation between any two observations decays with distance, here approaching zero at about 45 km. This is a short distance, certainly relative to the size of Ethiopia, so while we can see this short-range spatial pattern in the kriged map, the spatial pattern is seen at fine spatial scales and more detailed sampling would be needed to resolve it and to develop targeted interventions should these be desired. Figure 3b also shows us the kriging variance is very high in most parts of the country. A larger proportion of the total variance is in the uncorrelated "nugget" effects and spatial dependence is seen over shorter distances. As noted above, this does have implications for the potential to design interventions that are tailored to local conditions, for example agricultural interventions that might influence Zn status of crops in smallholder subsistence settings, including use of inputs such as manure and fertilizer^41^.

These new detailed analyses of the ENMS could inform programs or policies to improve surveillance and potentially alleviate Zn deficiency. The joint WHO/UNICEF/IAEA/IZiNCG meeting on Zn status indicators agreed that in populations where the Zn deficiency prevalence rate is > 20% it should be considered a public health concern, which is the case in Ethiopia^42^. Potential strategies to alleviate Zn deficiency include the promotion of dietary diversity and biofortification, i.e. the application of Zn to crops via fertilizers^43,44^ and the release of crop varieties containing higher concentrations of Zn^45^. The spatial analysis of these data show that, while there is spatial dependence in the biomarker, this is limited to short distances. This limits the value of spatial mapping for designing interventions to address Zn deficiency at national scale. However, it does suggest that, at local scale, there could be variations in the prevalence of Zn deficiency which mean that more efficient interventions could be better targeted on the basis of spatial information, based either on direct sampling of the biomarker or a better understanding of the environmental factors which drive the observed variability.

Strengths of our study include the large population coverage, comprising several demographic groups, using a cross-sectional sampling design. We improved on previous estimates of population Zn status by using more accurate and sensitive instrumental analyses to determine Zn concentration in serum samples, employing advanced geostatistical methods to predict the Zn status of the population across unsampled locations, and by adjusted serum Zn concentrations considering inflammation biomarkers using different cutoffs dependent on time of blood draw. However, in the ENMS, EAs are not evenly distributed between regions proportional to size of the population. In addition, in areas of small observations such as Afar, Somali and south-east Oromia regions the uncertainty of the prediction is large which warrants further sample analysis from the areas for predictive mapping work and estimating Zn nutrition status of the wider population. Rural electrification and access to roads is known to improve nutritional status through increasing employment, access to health care services, improving nutritional knowledge, reducing fertility, improved access to market, and creating wealth ^46,47^. Exclusion of EAs in the present study due to lack of access to road and electricity may underestimate the magnitude of national and regional Zn deficiency prevalence, suggesting the importance of further studies in the areas not covered in the present study. Analyses of potential contributors to variation in serum Zn in different areas and among different demographic groups, for example data on dietary intake, and drivers of variation in food composition such as soil, crop, and livestock Zn status, as reported previously for Se in Ethiopia^48^ is lacking in the present study. We are not therefore able to draw conclusions about the causal effect of low serum Zn. Different study designs will be required to identify explanatory factors contributing to the variation in Zn status and determine the health significance of the deficiencies observed in the current study.

## Conclusions

The present study shows that Zn deficiency is highly prevalent in Ethiopia, and provides the first estimation which accounts for the impact of inflammation on the assessment of Zn status in Ethiopia. Risk of Zn deficiency is spatially dependent within a short distance up to 45 km, which is likely to be due to environmental and food system factors including soil type, landscape features, and food production and distribution^11^. This study may inform national strategies to enhance Zn status of the Ethiopian population, and to improved micronutrient surveillance programs.

## Materials and methods

### Study design and sample population

**Figure 5:**
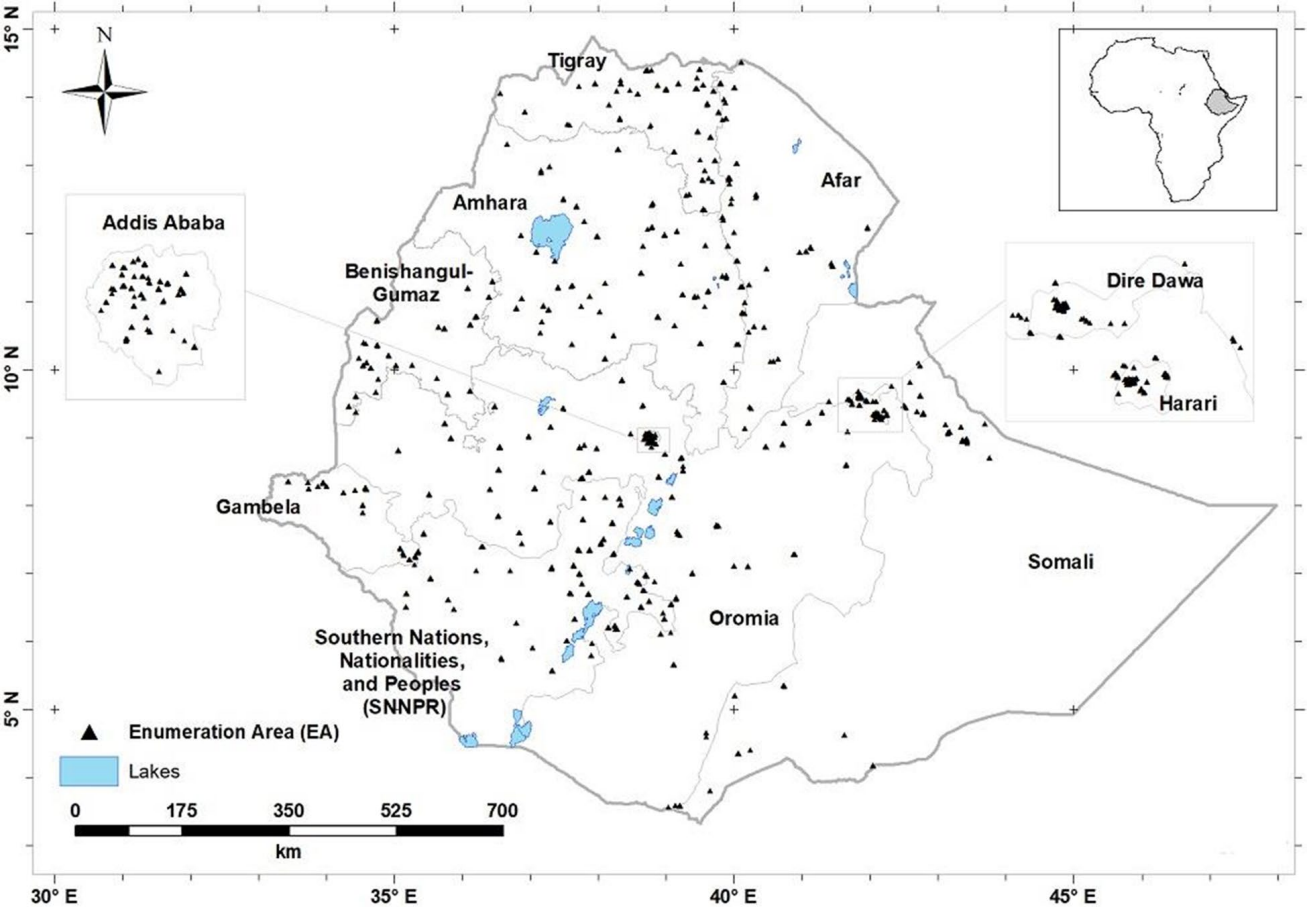
Locations of the centroids of n = 343 Enumeration Areas from which study participants were recruited^15^. Created using ArcGIS 10.4.1. ESRI ArcGIS Desktop: Release 10. Environmental Systems Research Institute, Redlands. (2011).

The ENMS covered all nine regions of Ethiopia and two administrative cities (Addis Ababa and Dire Dawa; Fig. 5). The design of the ENMS is explained in detail elsewhere^14,15,34^. Briefly, the ENMS was a large, population-based cross-sectional survey of Young Children (YC, aged 6–59 months, n = 1100), School Age Children (SAC, aged 5–15 years, n = 1500), Women of Reproductive Age (WRA, aged 15–49 years, n = 1600) and Men (aged 15–54 years, n = 500) conducted between March and July 2015. The ENMS enumeration areas (EAs), or clusters, are geographic areas defined by the Central Statistics Agency (CSA) for the Ethiopia Population and Housing Census^49^. The survey employed stratified sampling in each of the nine regions and two administrations cities. For each region, the EAs were selected based on standard probability proportional to size (PPS). The number of EAs among regions were adjusted at the sixth root for fair allocation of EAs especially for smaller regions. A total of 366 spatially distributed EAs were selected randomly. Following the household census conducted in all selected EAs preceding the actual survey, 13 of them were excluded due to lack of infrastructure (e.g. EAs that can’t be accessed by car or pack animals or are more than 5 h walking distance) and security issues. Within each selected EAs 11 households were randomly selected for enumeration. EAs contain on average 181 households (150–200)^49^.

The present study uses two datasets: socio-demographic data collected from georeferenced households in the ENMS, and newly-generated data on Zn concentrations measured in archived serum samples from the ENMS. The data on Zn concentrations were obtained only for those individuals from the ENMS survey for whom socio-demographic data, inflammation biomarker and location information from GPS were available, and where there was at least 0.3 mL of archived serum sample. A total of 3373 out of 4700 (71.7%) of the archived serum samples contained sufficient material for analysis of Zn concentration.

### Data collection and analysis

#### Socio-demographic data

Socio-demographic information was collected using a structured questionnaire. The research assistants and supervisors were trained on data collection. The questionnaire was pilot tested in a cluster that was not selected for the actual survey. Questionnaires were refined based on the finding from the pilot testing before the actual data collection began^15^.

#### Collection, processing and analysis of serum Zn

Blood collection and processing methods are described elsewhere^14,15,34^. Briefly, venous blood samples were collected from participants by trained phlebotomists and processed as per the World Health Organization (WHO) blood collection guidelines^50^. Samples were aliquoted in the field and subsequently analyzed for a range of micronutrients^14^. One aliquot of each sample was stored at EPHI at − 80 °C as archived material in case analyses needed to be repeated or additional micronutrient analyses could be supported, as is the case with the present study.

The concentration of Zn in serum samples was determined using ICP-MS (Thermo Fisher Scientific iCAPQ, Thermo Fisher Scientific, Bremen, Germany). Samples were introduced, via a single line, from an auto-sampler incorporating an ASXpress™ rapid uptake module (Cetac ASX-520, Teledyne Technologies Inc., Omaha, NE, USA) through a perfluoroalkoxy (PFA) Microflow PFA-ST nebulizer (Thermo Fisher Scientific, Bremen, Germany). All samples and external element calibration standards were diluted as 0.3 mL added to 6 mL of a solution containing: (1) 0.5% HNO_3_ (Primar Plus grade), (2) 2.0% methanol (Fisher Scientific UK Ltd, Loughborough, UK) and (3) three internal standards including ^72^Ge (10 µg L^−1^), ^103^Rh (5 µg L^−1^), ^193^Ir (5 µg L^−1^) (SPEX Certiprep Inc., Metuchen, NJ, USA). Zn calibration standards were prepared at 0, 20, 40, 100 µg L^−1^ (Claritas-PPT grade CLMS-2; SPEX Certiprep Inc., Metuchen, NJ, USA). The ICP-MS was operated in ‘collision-reaction cell mode’.

The limit of detection (LOD) for Zn was measured as 3 × standard deviation of 10 operational blanks; the limit of quantification (LOQ) was calculated as 10 × standard deviation. The LOD and LOQ were 1.43 and 4.77 µg L^−1^, respectively. Accuracy was verified by the use of two Seronorm™ reference materials: L-1 (Lot 1801802) and L-2 (Lot 1801803) (Nycomed Pharma AS, Billingstad, Norway). These were prepared in an identical way to the samples and typically run at the start and the end of each analytical run. Average Zn recovery (%; n = 24) when compared to accredited values determined across 10 analytical batches of blood serum was 88% and 86% for L-1 and L-2, respectively.

### Data analysis

Of the total of 3373 analyzed serum samples for Zn concentration, 309 observations were excluded due to missing GPS coordinates (n = 101; men = 5, YC = 63, SAC = 24 and WRA = 9), missing data on meal intake time (n = 172; men = 8. SAC = 38 and WRA = 126), missing inflammation biomarker (CRP and/or AGP) data (n = 30; WRA = 19, Men = 4 and SAC = 7) or a lack of socio-demographic data (n = 4; 2 from SAC and 2 from WRA) with two analytical outlier datum from WRA. Thus, 3064 observations were included for descriptive statistical analyses and considered for determination of prevalence of Zn deficiency. The mean, median and prevalence of Zn deficiency were determined before and after adjustment for inflammation.

Serum Zn concentrations were corrected for infection using the BRINDA internal regression approach^22^. This was done by running a linear regression model with lnZinc as the dependent variable; and lnCRP and lnAGP as the independent variables. This generated the slope (regression coefficient) of lnCRP (β1) and lnAGP (β2). These two parameters were then used to adjust for the effect of inflammation, for observations whose CRP and AGP values were above the reference values. A reference value for serum lnCRP and lnAGP is used to avoid over-adjusting the micronutrient biomarkers among individuals with low levels of inflammation. The reference values for this analysis were the maximum value of the lowest decile for lnCRP (p10) and that of lnAGP (p10).

The equation was thus, adjusted Zn concentration = exp [unadjusted lnZinc– β1(CRPobserved – maximum of lowest decile for CRP) – β2 (AGPobserved – maximum of lowest decile for AGP)]. The model was checked to ensure all assumptions were met by examining the plot of residuals, homogeneity of variance, and normality.

Descriptive statistical analyses were conducted in STATA (Version 14.0, StataCorp LLP, Texas, USA). Prevalence of Zn deficiency among all demographic groups in Ethiopia were determined as suggested by Hotz et al. 2003^33^. Zn deficiency defined as serum Zn concentration < 65 µg dL^−1^ for morning, non-fasting samples for YC and SAC under 10 years of age and < 57 µg dL^−1^ for afternoon, non-fasting samples. For those 10 years old and older, a cutoff of < 70 µg dL^−1^ was used for males and < 66 µg dL^−1^ was used for females for morning, non-fasting samples; the cutoff of < 61 µg dL^−1^ was used for males and < 59 µg dL^−1^ was used for females for afternoon, non-fasting samples. A cutoff of < 74 µg dL^−1^ was used for men and < 70 µg dL^−1^ was used for WRA for morning, fasting samples^10^.

Serum Zn concentrations were mapped at national level for WRA only. This is because WRA had the largest sample size in the ENMS and the sample covered more EAs than participants of other demographic groups in the survey. Inclusion of all age categories in the modelling could have clustering effect causing a non-reliable predictive mapping, and would have limited our scope to compare to appropriate biomarker thresholds which are specific to demographic groups due to the variation in concentration expected with age. Even though serum zinc concentration of the present study participants differs by age category, it isn’t expected that the pattern of serum zinc concentration distribution to be different among these groups. This is because the study participants were either from the same households or villages dependent on local food sources. For example, our previous studies show that the spatial pattern of distribution of serum Se concentration among women of reproductive age and young children in the Amhara region was not different^15,51^. Ordinary kriging predictions were computed on the nodes of a 60-m square grid. In geostatistical terminology, a prediction is obtained from the prediction distribution of a random variable at an unsampled location. This is a conditional distribution, conditional on the geostatistical model (the variogram in the case of ordinary kriging) and the observed values at sample locations. In ordinary kriging, the prediction is the mean of the prediction distribution, and its variance is the ordinary kriging variance.

Data values were first aggregated to mean values for each EA, and mean coordinates were computed for each EA. One single value was a probable outlier according to the criteria of Tukey (1977)^25^. This was removed from the data for variogram estimation but was returned for spatial prediction by kriging. This is because estimates of the variogram are particularly susceptible to extreme values^26^ but if, as here, there is no reason to think the observation erroneous then it should be considered for local prediction.

Because of the large extent of the sampled region, all spatial analyses were done using the latitude and longitude recorded for the observations, rather than on a rectilinear grid since no single projection would be suitable at all locations. Under the usual assumptions of stationarity in geostatistics, spatial dependence is modelled in terms of the vector that separates locations, or lag. In this setting, the lag distance between any two points was computed as the great circle distance on a spherical approximation. This was computed using the *dist Vincenty Sphere* function from the geosphere package for the R platform^52^. The function *final Bearing* from the same package was used to obtain bearings. Note that, when distances between locations are measured on the sphere, then not all variogram functions that are suitable for use on the plane can be applied. On the sphere the exponential model is authorized^53^, and so it was used for all variogram modelling in this study.

Exploratory variogram analysis indicated that there was no marked directional dependence of the variogram (Supplementary Figure S1), so an isotropic variogram (Supplementary Figure S2) was estimated using the standard estimator due to Matheron (1962)^23^. In addition, variogram values were estimated using the robust estimators due to Dowd (1984)^54^ and to Cressie and Hawkins (1980)^55^. These alternative estimators were considered because, although a marginal outlier (an unusual datum as it appears on the overall distribution) was removed, spatial outlier(observations unusual in its spatial context) can inflate estimates of the semivariance^26^. An exponential variogram model^24^ was fitted by weighted least squares, and then tested by cross-validation. In cross-validation each observation was, in turn, removed and predicted from the remaining observations by ordinary kriging. The squared cross-validation error at each site was standardized by dividing by the ordinary kriging variance. The median standardized squared prediction error (SSPE) has an expected value of 0.455 in the case of a valid underlying variogram model with kriging errors that appear normally distributed^25^. The estimator due to Matheron (1962)^23^ is more statistically efficient than the robust alternatives, so if the model fitted to these estimates appeared correct from the cross-validation results (median SSPE) then the alternatives were not considered. If the SSPE suggests that the model fitted to Matheron estimates are affected by outliers, then the models fitted to robust estimates are also cross-validated, and one is selected on the cross-validation results. This procedure follows the recommendations of Lark (2000)^26^.

Our spatial analysis is focused on WRA because this was the demographic group with the largest sample size with coverage of 315 out of 343 enumeration areas. The statistical summary for WRA is shown in Supplementary Figure S3.

### Ethical approval

This study was conducted according to the national research ethics guideline by the Ethiopian Science and Technology Ministry. All procedures involving human subjects were approved by the National Health Research Ethics Review Committee at the Ethiopian Science and Technology Ministry (Reference 3.10/433/06) and support letters were obtained from the respective regional and local health bureaus. Adult participants gave informed consent for their participation. Informed consent was also obtained from mothers or guardians for their children to participate in the study. In addition, assent was obtained from participating school age children. A separate ethical approval was obtained for the present study from the Research Ethical Review Committee of the Ethiopian Public Health Institute (Protocol EPHI-IRB-140-2018). Archived serum samples were transferred from storage at EPHI to the University of Nottingham, UK for analysis under a Material Transfer Agreement.

## Supplementary Information

Supplementary Information 1.

Supplementary Information 2.

## Data Availability

The data generated in this study is available based on acceptable reasoning from the corresponding authors.
