# Supplementary material for: Zinc deficiency is highly prevalent and spatially dependent over short distances in Ethiopia
Source: Sci Rep. 2021 Mar 22;11:6510. doi: 10.1038/s41598-021-85977-x (PMC7985319; doi:10.1038/s41598-021-85977-x)
Supplement: Supplementary file 1 — Supplementary Information 1. [file 41598_2021_85977_MOESM1_ESM.doc]

**Zinc deficiency is highly prevalent and spatially dependent over short distances in Ethiopia**

Adamu Belay^1,2^, Dawd Gashu^1*^, Edward J. M. Joy^3^, R. Murray Lark^4^, Christopher Chagumaira^4^, Blessings H. Likoswe^5^,Dilnesaw Zerfu^2^, E. Louise Ander^6^, Scott D. Young^4^, Elizabeth H. Bailey^4^& Martin R. Broadley^4^

^1^ Center for Food Science and Nutrition, Addis Ababa University, P. O. Box 1176, Addis Ababa, Ethiopia

^2^ Food Science and Nutrition Research Directorate, Ethiopian Public Health Institute, Gulele Sub City, P.O.Box 1242 Addis Ababa, Ethiopia.

^3^Faculty of Epidemiology and Population Health, London School of Hygiene & Tropical Medicine, Keppel Street, London, WC1E 7HT, UK.

^4^School of Biosciences, University of Nottingham, Sutton Bonington Campus, Loughborough, Leicestershire, LE12 5RD, UK.

^5^Department of Public Health, School of Public Health and Family Medicine, College of Medicine, University of Malawi, Private Bag 360, Chichiri, Blantyre 3, Malawi

^6^Inorganic Geochemistry, Centre for Environmental Geochemistry, British Geological Survey, Nottingham, NG12 5GG, UK.

**Corresponding author*: Dr. Dawd Gashu; Center for Food Science and Nutrition, Addis Ababa University; [dawd.gashu@aau.edu.et](mailto:dawd.gashu@aau.edu.et); P. O. Box 1176

Supplementary Table S1**.** Serum Zn concentrations and prevalence of deficiency in Ethiopia Zonal Administration, 2016

| Characteristics | Serum Zn (µg dL^-1^) | | | | | Prevalence of Zn deficiency (%) | |
| --- | --- | --- | --- | --- | --- | --- | --- |
|  | Unadjusted | | | Adjusted | |  |  |
| Regions and Zone | n | Mean | Median | Mean | Median | Unadjusted | Adjusted |
| **Afar** | 270 | 59.2 | 58.6 | 60.1 | 59.4 | 63.2 | 61.0 |
| Awsi Rasu | 50 | 62.15 | 61.69 | 62.74 | 62.39 | 53.25 | 53.25 |
| Kilbet Rasu | 113 | 57.86 | 57.64 | 58.78 | 58.45 | 68.49 | 65.87 |
| Gabi Rasu | 20 | 61.43 | 59.57 | 62.32 | 61.88 | 57.87 | 57.87 |
| Fanti Rasu | 45 | 63.88 | 65.18 | 65.36 | 65.18 | 36.90 | 30.46 |
| Hari Rasu | 42 | 54.19 | 52.25 | 55.27 | 52.61 | 80.74 | 77.42 |
| **Amhara** | 481 | 57.6 | 57.4 | 58.6 | 58.1 | 74.1 | 71.5 |
| Awi | 29 | 56.60 | 58.74 | 57.83 | 59.01 | 82.01 | 78.53 |
| East Gojjam | 50 | 59.47 | 57.77 | 60.26 | 58.99 | 67.03 | 64.84 |
| North Gondar | 93 | 57.32 | 57.11 | 58.55 | 58.00 | 72.09 | 67.74 |
| North Shewa | 38 | 54.84 | 55.37 | 55.49 | 56.14 | 87.50 | 81.58 |
| North Wello | 72 | 58.41 | 57.79 | 59.45 | 58.71 | 63.47 | 62.03 |
| Kemissie | 6 | 51.29 | 48.36 | 52.09 | 48.90 | 83.33 | 83.33 |
| South Gondar | 61 | 57.23 | 55.53 | 58.46 | 56.34 | 76.48 | 69.45 |
| South Wello | 64 | 54.69 | 53.83 | 55.39 | 55.01 | 80.16 | 77.22 |
| West Gojjam | 68 | 57.94 | 59.02 | 59.07 | 60.37 | 75.99 | 75.99 |
| **Benishangul-Gumuz** | 210 | 57.7 | 57.6 | 58.9 | 58.5 | 69.9 | 65.7 |
| Asosa | 71 | 58.58 | 58.06 | 59.75 | 59.99 | 71.55 | 68.29 |
| Kamashi | 28 | 55.65 | 54.37 | 56.96 | 55.26 | 78.29 | 75.00 |
| Mao Komo Special | 34 | 55.31 | 52.00 | 56.75 | 55.71 | 76.64 | 73.49 |
| Metekel | 64 | 58.90 | 61.28 | 60.06 | 61.87 | 61.19 | 54.18 |
| Pawe special | 13 | 58.05 | 56.72 | 59.09 | 58.37 | 69.23 | 69.23 |
| **Dire Dawa** | 160 | 60.9 | 60.8 | 61.9 | 61.7 | 66.4 | 64.0 |
| **Gambela** | 192 | 59.3 | 58.3 | 60.4 | 59.3 | 64.5 | 63.2 |
| Agnewak | 53 | 55.83 | 56.19 | 56.71 | 57.21 | 83.34 | 83.63 |
| Etang special | 13 | 59.06 | 61.84 | 60.53 | 62.91 | 69.23 | 61.54 |
| Mejenger | 86 | 59.19 | 57.32 | 60.31 | 59.34 | 60.74 | 58.55 |
| Nuer | 40 | 65.07 | 62.36 | 66.14 | 62.44 | 43.54 | 43.78 |
| **Harari** | 168 | 56.4 | 55.7 | 57.3 | 56.9 | 71.8 | 69.7 |
| **Oromia** | 517 | 54.8 | 54.5 | 56.1 | 55.6 | 80.0 | 76.7 |
| Adama special | 12 | 71.63 | 72.77 | 72.44 | 74.03 | 25.00 | 25.00 |
| Arsi | 45 | 58.67 | 58.01 | 60.00 | 59.95 | 77.22 | 72.67 |
| Bale | 21 | 58.99 | 58.44 | 59.45 | 58.41 | 61.71 | 61.71 |
| Borena | 7 | 56.88 | 56.05 | 59.37 | 63.21 | 85.71 | 71.43 |
| East Hararge | 61 | 47.45 | 45.64 | 48.67 | 46.63 | 93.64 | 90.06 |
| East Shewa | 24 | 60.56 | 59.27 | 61.81 | 60.44 | 60.97 | 52.76 |
| East Wellega | 25 | 55.35 | 55.45 | 56.775 | 55.61 | 75.91 | 72.74 |
| Guji | 26 | 55.24 | 54.01 | 56.45 | 54.70 | 83.58 | 80.39 |
| Horo Gudru Wellega | 16 | 53.53 | 54.34 | 55.07 | 56.48 | 87.50 | 81.25 |
| Ilu Aba Bora | 39 | 52.03 | 53.77 | 53.23 | 54.31 | 94.52 | 89.37 |
| Jimma | 49 | 52.81 | 53.10 | 54.29 | 54.31 | 85.46 | 83.52 |
| Kelem Wellega | 19 | 51.50 | 51.40 | 52.95 | 51.91 | 78.95 | 78.95 |
| North Shewa | 14 | 54.50 | 55.31 | 55.62 | 56.12 | 87.50 | 81.58 |
| South-west Shewa | 25 | 50.09 | 49.04 | 50.77 | 49.39 | 80.16 | 77.22 |
| West Arsi | 33 | 57.10 | 56.64 | 58.61 | 59.52 | 83.29 | 78.89 |
| West Hararge | 40 | 56.49 | 56.72 | 57.51 | 58.62 | 71.22 | 68.49 |
| West Shewa | 33 | 60.85 | 60.15 | 62.71 | 62.82 | 60.46 | 60.48 |
| West Wellega | 28 | 56.58 | 59.39 | 58.38 | 60.25 | 79.06 | 78.44 |
| **SNNPR** | 392 | 57.0 | 57.3 | 58.4 | 58.8 | 73.3 | 69.8 |
| Alaba special | 6 | 55.73 | 49.49 | 56.67 | 50.70 | 83.33 | 83.33 |
| Amaro Special | 14 | 62.70 | 61.99 | 64.70 | 64.90 | 28.57 | 21.43 |
| Bench Maji | 24 | 57.23 | 56.88 | 58.91 | 58.14 | 70.64 | 70.64 |
| Dawuro | 12 | 59.24 | 57.80 | 60.15 | 58.50 | 75.00 | 66.67 |
| Derashe special | 6 | 47.74 | 45.19 | 49.12 | 47.15 | 83.33 | 83.33 |
| Gamo Gofa | 32 | 55.40 | 54.90 | 56.61 | 55.33 | 76.01 | 66.82 |
| Gedeo | 15 | 58.40 | 59.94 | 59.37 | 61.30 | 73.48 | 73.48 |
| Gurage | 24 | 60.65 | 57.31 | 62.32 | 58.91 | 67.49 | 68.69 |
| Hadiya | 47 | 54.66 | 54.21 | 55.92 | 55.02 | 85.21 | 80.46 |
| Hawassa city Adm. | 9 | 66.21 | 65.74 | 66.92 | 66.38 | 55.56 | 55.56 |
| Kefa | 18 | 56.75 | 57.66 | 58.08 | 59.95 | 72.23 | 72.23 |
| Kembata Tibaro | 23 | 57.00 | 54.50 | 58.59 | 56.70 | 78.88 | 78.88 |
| Sheka | 13 | 55.73 | 53.90 | 56.95 | 55.58 | 84.62 | 76.92 |
| Sidama | 67 | 56.02 | 58.55 | 57.42 | 59.35 | 77.87 | 75.00 |
| Siltie | 24 | 53.43 | 53.65 | 55.27 | 56.87 | 82.49 | 75.25 |
| South Omo | 9 | 61.53 | 57.71 | 63.00 | 58.37 | 77.78 | 77.78 |
| Wolayita | 49 | 58.69 | 60.64 | 60.03 | 61.88 | 60.11 | 56.78 |
| **Somali** | 193 | 60.5 | 59.7 | 61.8 | 60.9 | 66.5 | 63.8 |
| Jijiga | 95 | 63.52 | 62.25 | 64.49 | 62.90 | 61.34 | 57.70 |
| Liben | 70 | 56.91 | 56.26 | 57.94 | 56.80 | 72.61 | 70.14 |
| Shinile | 28 | 58.77 | 55.54 | 59.64 | 55.22 | 69.68 | 69.68 |
| **Tigray** | 333 | 54.8 | 54.0 | 55.6 | 55.2 | 78.5 | 75.5 |
| Central Tigray | 80 | 51.91 | 51.71 | 52.76 | 52.39 | 87.91 | 83.95 |
| Eastern Tigray | 67 | 57.92 | 56.59 | 58.88 | 57.92 | 68.98 | 66.08 |
| Mekele town special | 20 | 60.08 | 61.68 | 60.91 | 62.41 | 45.07 | 45.07 |
| North-west Tigray | 32 | 53.30 | 52.48 | 54.02 | 53.31 | 86.79 | 80.92 |
| Southern Tigray | 114 | 54.19 | 53.24 | 54.88 | 53.76 | 80.13 | 77.54 |
| Western Tigray | 20 | 55.88 | 54.99 | 57.37 | 55.44 | 85.88 | 85.88 |
