# Supplementary material for: Zinc deficiency is highly prevalent and spatially dependent over short distances in Ethiopia
Source: Sci Rep. 2021 Mar 22;11:6510. doi: 10.1038/s41598-021-85977-x (PMC7985319; doi:10.1038/s41598-021-85977-x)
Supplement: Supplementary file 2 — Supplementary Information 2. [file 41598_2021_85977_MOESM2_ESM.docx]

**Zinc deficiency is highly prevalent and spatially dependent over short distances in Ethiopia**

Adamu Belay^1,2^, Dawd Gashu^1*^, Edward J. M. Joy^3^, R. Murray Lark^4^, Christopher Chagumaira^4^, Blessings H. Likoswe^5^,Dilnesaw Zerfu^2^, E. Louise Ander^6^, Scott D. Young^4^, Elizabeth H. Bailey^4^& Martin R. Broadley^4^

^1^ Center for Food Science and Nutrition, Addis Ababa University, P. O. Box 1176, Addis Ababa, Ethiopia

^2^ Food Science and Nutrition Research Directorate, Ethiopian Public Health Institute, Gulele Sub City, P.O.Box 1242 Addis Ababa, Ethiopia.

^3^Faculty of Epidemiology and Population Health, London School of Hygiene & Tropical Medicine, Keppel Street, London, WC1E 7HT, UK.

^4^School of Biosciences, University of Nottingham, Sutton Bonington Campus, Loughborough, Leicestershire, LE12 5RD, UK.

^5^Department of Public Health, School of Public Health and Family Medicine, College of Medicine, University of Malawi, Private Bag 360, Chichiri, Blantyre 3, Malawi

^6^Inorganic Geochemistry, Centre for Environmental Geochemistry, British Geological Survey, Nottingham, NG12 5GG, UK.

**Corresponding author*: Dr. Dawd Gashu; Center for Food Science and Nutrition, Addis Ababa University; [dawd.gashu@aau.edu.et](mailto:dawd.gashu@aau.edu.et); P. O. Box 1176

Supplementary Figure S1: Directional-dependent estimates of the variogram

Supplementary Figure S2: Estimates of the isotropic variogram


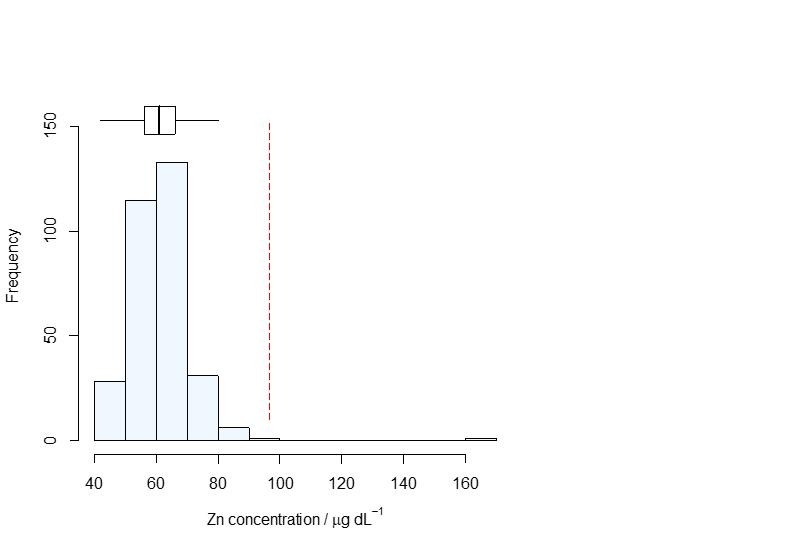


Supplementary Figure S3: Summary statistics for serum zinc concentration among women of reproductive age
